# Olfactory and gustatory dysfunction in COVID‐19 patients: A meta‐analysis study

**DOI:** 10.14814/phy2.14578

**Published:** 2020-09-25

**Authors:** Bahareh Hajikhani, Tess Calcagno, Mohammad Javad Nasiri, Parnian Jamshidi, Masoud Dadashi, Mehdi Goudarzi, Adrien A. Eshraghi, FACS, Mehdi Mirsaeidi

**Affiliations:** ^1^ Department of Microbiology School of Medicine Shahid Beheshti University of Medical Sciences Tehran Iran; ^2^ Miller School of Medicine University of Miami Miami FL USA; ^3^ Student Research Committee, School of Medicine Shahid Beheshti University of Medical Sciences Tehran Iran; ^4^ Department of Microbiology School of Medicine Alborz University of Medical Sciences Karaj Iran; ^5^ Department of Otolaryngology University of Miami Hearing Research Laboratory Miller School of Medicine Miami FL USA; ^6^ Division of Pulmonary and Critical Care University of Miami Miami FL USA

**Keywords:** COVID‐19, gustatory, olfactory, smell, taste

## Abstract

COVID‐19, caused by a novel coronavirus, is a persistent global pandemic. It is crucial to examine existing reports to effectively summarize and characterize its clinical course. We used a large‐scale meta‐analysis to establish prevalence rates for loss of olfaction and gustation in COVID‐19 positive patients. PubMed/Medline, Embase, and Web of Sciences were searched for articles published until April 30, 2020. Furthermore, to avoid missing papers, more searches were carried out in the reference lists of covered studies. Articles that mentioned olfactory and/or gustatory disorder in patients with COVID–19 were included for further analysis. Articles that did not report the aforementioned information were excluded. Duplicated articles, reviews, and meta‐analysis were excluded as well. The quality of the references was assessed according to the checklist provided by JBI (Joanna Briggs Institute). We used independent extraction of data by multiple observers. The pooled frequency with 95% confidence intervals (CI) was assessed using random effect model. The main outcome measures reported were the pooled frequency of olfaction and pooled frequency of gustation disorder in patients with COVID‐19 calculated using a random effect model weighted by the study population. The 15 included studies had 3,739 participants which all had confirmed COVID–19. Olfactory and gustatory disorders were assessed and a total number of 1,354 and 1,729 were reported to have taste or smell impairment, respectively. The estimated rate of taste disorder in patients with COVID‐19 was 49.0% [95% confidence interval (CI) 34.0–64.0, I2: 96%] (Figure 2). The estimated rate of olfactory disorder in patients with COVID‐19 was 61.0% (95% CI 44.0%–75.0%). Our meta‐analysis demonstrated high rates of taste (49.0%) and smell (61.0%) disorders in patients with confirmed COVID‐19. Results increase the power of recent reports—loss of olfactory and loss of gustation should now routinely be considered in the setting of COVID‐19 infection.

## Graphical abstract

Impaired olfaction and gustation should be used to aid in the early diagnosis and effective viral containment of COVID‐19. The estimated prevalence rate of taste disorder was 49%in patients with confirmed COVID‐19. The estimated prevalence rate of smell disorder was 61%in patients with confirmed COVID‐19.

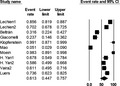

## 1 INTRODUCTION

A viral outbreak caused by a novel coronavirus (SARS‐CoV2) emerged from Wuhan, China in late December 2019 (Zhu et al., 2020). Within three months, the disease caused by SARS‐CoV2 (COVID‐19), quickly became a global pandemic and to date has caused over 270 thousand deaths worldwide. Health authorities have been working together to determine future spread of the virus, streamline antiviral medications, develop a functional vaccine, and better define its clinical course.

COVID‐19 is known for causing acute respiratory syndrome with nonspecific presenting symptoms characterized by fever, cough, chills, dyspnea, myalgia, and sore throat (Tahvildari et al., 2020). However, COVID‐19 can also cause loss of smell (anosmia) and loss of taste (ageusia) early in the disease. In fact, there are some reports that COVID‐19 patients experience a period of anosmia and/or ageusia without any other symptoms (Hummel, Landis, & Huttenbrink, 2011; Karimi‐Galougahi, Raad, & Mikaniki, 2020). Olfactory and taste dysfunction have very recently been reported to be higher in home‐quarantined, young, and female patients (Paderno et al., 2020). Screening for loss of taste or smell in otherwise asymptomatic individuals may be an effective strategy to stop transmission early in the disease. Additionally, the presence of anosmia and/or ageusia in a patient presenting with nonspecific respiratory symptoms could help to rule out infection with influenza in the upcoming flu season. Although olfactory and gustation dysfunctions have been reported in a few studies, prevalence rates have not been evaluated in a large sample size meta‐analysis. We conducted a systematic review and meta‐analysis to define the prevalence of these unique symptoms among confirmed COVID‐19 subjects.

## 2 MATERIALS AND METHODS

This study was conducted and reported according to the PRISMA guidelines (Moher, Liberati, Tetzlaff, & Altman, 2009). The study was the **Systematic Review Registration**: PROSPERO (pending registration ID: 187697).

### 2.1 Search strategy

To identify potentially applicable studies, the three most important electronic databases encompass PubMed/Medline, Embase, and Web of Sciences were searched for articles published until May 2, 2020. Furthermore, to avoid missing papers, more searches were carried out in the reference lists of covered studies. The current study did not require any ethics committee approval.

The search keywords used were as follow: “COVID–19,” “Novel coronavirus 2019,” “2019 nCoV,” and “SARS‐CoV‐2” as well as “smell disorder,” “taste disorder,” and their similar terms such as “olfactory,” “dysosmia,” “anosmia,” “gustatory,” “ageusia,” and “dysgeusia.” Studies in English were included. Two different investigators independently evaluated search results.

### 2.2 Inclusion and exclusion criteria

Any applicable articles that mentioned olfactory and/or gustatory disorder in patients with approved COVID–19 were included for further analysis. Articles which not reported aforementioned information were excluded. Duplicated articles, reviews, and meta‐analysis were excluded as well.

### 2.3 Data extraction and quality assessment

Two researchers designed a data extraction form to import data from all eligible studies. We extracted the following variables: first author name; year of publication; type of study, country where the study was performed; age and gender distribution, number of patients with confirmed COVID–19, number of patients with olfactory and/or gustatory disorder, type of olfactory and/or gustatory disorder. The quality of the references was assessed according to the checklist provided by JBI (Joanna Briggs Institute) (Munn, Moola, Lisy, & Riitano, 2014).

### 2.4 Statistical analysis

Statistical analyses were performed with STATA (version 14, IC; Stata Corporation, College Station, TX, USA). The pooled frequency with 95% confidence intervals (CI) was assessed using the random effect model. The between‐study heterogeneity was assessed by Cochran's Q and the I2 statistic. To explore heterogeneity, subgroup analyses stratified by disease type were performed. Publication bias was assessed statistically by Begg's test (*p* < .05 was considered indicative of statistically significant publication bias).

## 3 RESULTS

**FIGURE 1:**
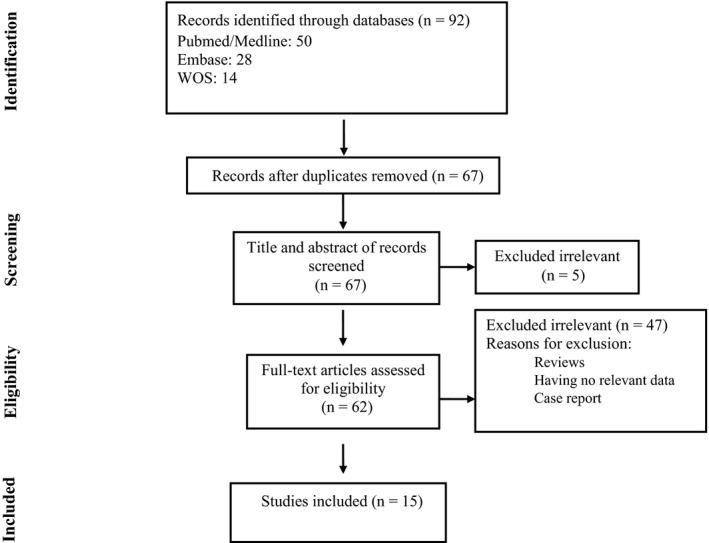
Flow chart of study selection for inclusion in the systematic review and meta‐analysis

The preliminary 92 citations had been recognized based on the evaluation of the title and abstract of the literature. Of them, 30 articles did not meet the inclusion criteria, including duplicated studies, case reports, articles without related data, review, and meta‐analysis publication. After reviewing the full text of 62 studies, 15 studies were selected for further assessment (Figure 1). Based on the JBI, the included papers had a low risk of bias.

Included studies were published between April 2020 and May 2020. The 15 included studies had 3,739 participants which all had confirmed COVID–19. The mean age of patients varied from 34 to 65 years. The olfactory and gustatory disorders were assessed and a total number of 1,354 and 1729 were reported to have taste or smell impairment, respectively. The evaluated patients in selected articles had mild to moderate infections and were treated as outpatients (Table 1).

**TABLE 1:** Characteristics of the included studies

First author	Published time	Country	Total No. of patients	No. of Men	No. of Women	Mean age	COVID−19 confirmation test
Lechien, Chiesa‐Estomba, De Siati, et al. (2020))	April 8 2020	Multicenter	417	154	263	37	RT‐PCR
Lechien, Chiesa‐Estomba, Place, et al. (2020))	May 1 2020	Multicenter	1,420	458	962	39	RT‐PCR
Beltran‐Corbellini et al. (2020)	April 23 2020	Spain	79	48	31	61	RT‐PCR
Benezit et al. (2020)	April 19 2020	France	68				RT‐PCR
Giacomelli et al. (2020)	April 7 2020	Italy	59	40	19	60	RT‐PCR
Klopfenstein et al. (2020)	April 28 2020	France	54	18	36	47	RT‐PCR
Mao et al. (2020)	April 24 2020	China	214	87	127	52	RT‐PCR
Moein et al. (2020)	April 28 2020	Iran	60	40	20	46	RT‐PCR
Spinato et al. (2020)	April 23 2020	Italy	202	97	105	56	RT‐PCR
Wee et al. (2020)	April 25 2020	Singapore	154				RT‐PCR
Yan, Faraji, Prajapati, Ostrander, Faraji, Prajapati, Ostrander, and DeConde (2020))	April 22 2020	USA	59	30	29		RT‐PCR
Yan, Faraji, Prajapati, Ostrander, et al. (2020))	April 25 2020	USA	128	61	67	34–65	RT‐PCR
Vaira, Salzano, et al. (2020))	May 2 2020	Italy	33	11	22	47	RT‐PCR
Vaira, Deiana, et al. (2020))	April 29 2020	Italy	72	27	45	49	RT‐PCR
Luers et al. (2020)	May 2 2020	Germany	72	41	31	38	RT‐PCR

### 3.1 Prevalence of gustatory disorder

**FIGURE 2:**
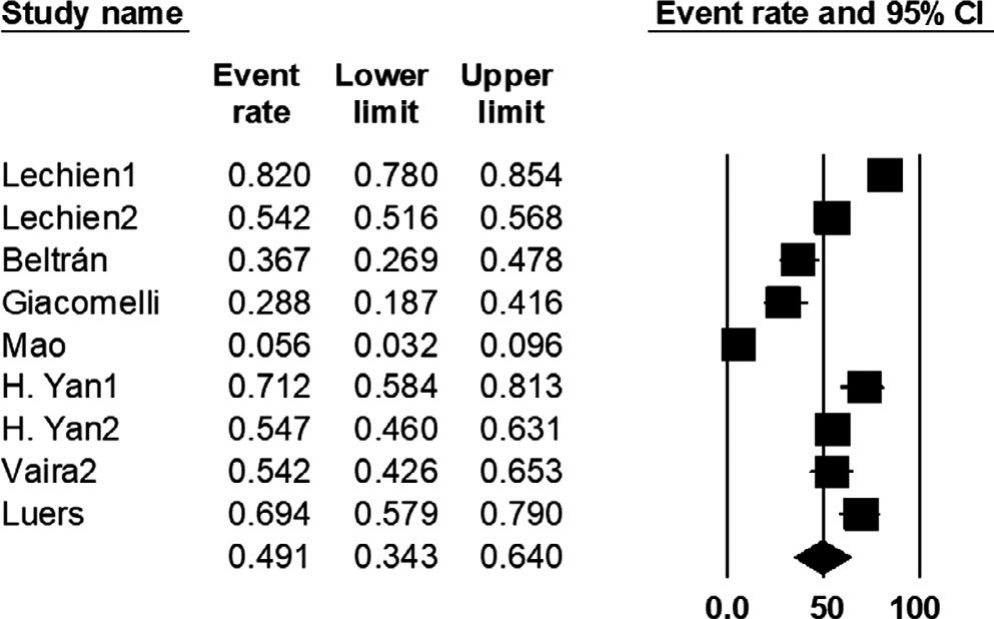
The pooled prevalence of taste disorder in patients with COVID‐19. Effects and summaries were calculated using a random effect model weighted by the study population

The estimated rate of gustatory disorder in patients with COVID‐19 was 49.1% [95% confidence interval (CI) 34.3–64.0, I2: 96%] (Figure 2). Based on Begg's test, publication bias was not observed in the induced studies (*p*‐value: .53).

### 3.2 Prevalence of olfactory disorder

**FIGURE 3:**
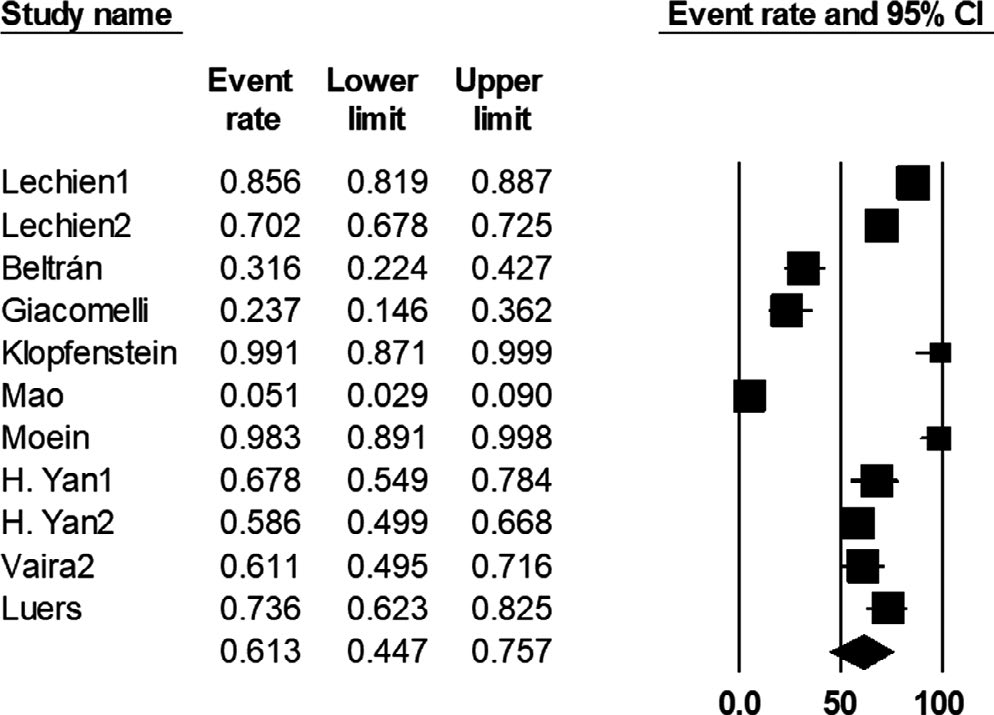
The pooled prevalence of smell disorder in patients with COVID‐19. Effects and summaries were calculated using a random effects model weighted by the study population

The estimated prevalence rate of olfactory disorder in patients with COVID‐19 was 61.3% (95% CI 44.7–75.7, I2: 95%) (Figure 3). No evidence of publication bias was observed (Begg's tests *p*‐value was .60).

### 3.3 Subgroup analysis

Table 2 shows the subgroup analysis of the studies based on anosmia, hyposmia, hypogeusia, dysgeusia, and ageusia.

**TABLE 2:** Subgroup analysis

Groups	Number of studies	Frequency % (95% CI)	*n*/N*	Publication bias (*p‐*value)	Heterogeneity test	
					I^2^ (%)	*p‐*value
Anosmia	8	32.5 (14.0–58.8)	420/833	0.90	95	0.00
Hyposmia	7	44.7 (20.0–72.5)	263/788	0.88	97	0.00
Hypogeusia	6	45.0 (22.8–70.0)	398/728	0.45	95	0.00
Dysgeusia	3	23.0 (5.0–60.0)	87/266	0.30	95	0.00
Ageusia	6	16.4 (7.0–35.0)	139/719	0.45	93	0.00

## 4 DISCUSSION

This study found that the estimated prevalence rates of smell disorder were 61% and the taste disorder was 49% in patients with confirmed COVID‐19. Subgroup analysis defined the degree of impairment in olfactory and gustatory symptoms. Hyposomnia was more common than a complete loss of smell. Hypogeusia and dysgeusia were more common than a complete loss of taste.

Gustatory and olfactory disorders are recently accepted as COVID‐19 symptoms and have been reported with relatively high frequencies from all around the world (Tong, Wong, Zhu, Fastenberg, & Tham, 2020). According to our study, varying degrees of dysfunction exist indicating a potentially progressive pattern.

Viral upper respiratory tract infections are commonly associated with transient loss of smell and taste secondary to mucus accumulation (Soler, Patel, Turner, & Holbrook, 2020). If symptoms do not improve, patients can develop post‐viral olfactory disorder (PVOD) secondary to sensorineural insult. The University of Cincinnati Taste and Smell Center reported preceding viral infection in 22% of patients presenting with anosmia and the University of Pennsylvania reported preceding viral infection in 26% (Seiden, 2004). Suzuki *et al*. collected nasal discharge from 24 patients with PVOD and identified human rhinovirus, coronavirus, parainfluenza virus, and Epstein–Barr virus strains (Suzuki et al., 2007). However, it has been difficult to identify specific viral etiologies of PVOD since patients often present after the resolution of primary infection.

Various theories to explain the pathophysiology of viral smell and taste dysfunction have been purposed, but still, need to be clearly elucidated. It is possible that nasal congestion and rhinorrhea blunt sensory input to neurons carrying smell and taste; however, some COVID‐19 positive patients experience a period of anosmia and/or ageusia without any other symptoms (Hummel et al., 2011; Karimi‐Galougahi et al., 2020). Direct involvement with the peripheral or central nervous system is more plausible and is supported by studies performed on SARS coronavirus during its epidemic in 2002–2003 (Hwang, 2006; Xu et al., 2005).

Like other viruses in its family, SARS‐CoV2 probably gains entry into the nervous system via the olfactory bulb (Desforges, Le Coupanec, Brison, Meessen‐Pinard, & Talbot, 2014; Gu et al., 2005; Netland, Meyerholz, Moore, Cassell, & Perlman, 2008; Wu et al., 2020). Sustentacular cells are supporting cells for the olfactory neurons residing on the olfactory epithelium. They are lined with ACE‐2 and TMPRSS‐2 receptors used by SARS‐CoV2 for cellular entry. Disturbances in olfaction may involve sustentacular neuronal support cells rather than olfactory neurons which have a much lower expression of ACE‐2 and TMPRSS‐2 receptors (Bilinska, Jakubowska, Von Bartheld, & Butowt, 2020; Qi, Qian, Zhang, & Zhang, 2020).

After entry into sustentacular cells, SARS‐CoV2 may cause persistent damage through stem cell alterations. Regeneration in the olfactory neuroepithelium requires functioning neuronal stem cells. Horizontal basal stem cells (HBCs) and globose basal stem cells (GBCs) are responsible for olfactory regeneration. GBCs proliferate in uninjured olfactory epithelium, while HBCs proliferate in response to sustentacular cell death (Schwob et al., 2017). In chronic states of inflammation, HBC regeneration is impaired. In response to NF‐κB, a chemokine released during chronic inflammation, the function of HBCs is changed. They no longer function as stem cells and instead they amplify inflammatory signaling. Mouse studies of HBCs show an NF‐κB‐dependent upregulation of cytokines (CCL10, CCL20, and CXCL10) (Chen, Reed, & Lane, 2019).

Gustatory dysfunction is likely secondary to the loss of smell since there is no direct or indirect damage to taste buds or related neurons (Deems et al., 1991; Moein et al., 2020). This is plausible according to the findings from our study which showed lower rates of taste disorder as compared to smell disorder.

### 4.1 Limitations and strengths

Our paper is the first large‐scale analysis to report the prevalence of olfactory and gustatory abnormalities in patients with confirmed COVID‐19, symptoms which were newly reported in April 2020. The sample size was large, including 15 studies with 3,739 subjects. Results can also be applied to a large cohort of patients since patient age varied from 34 to 65 years. However, we did not study symptom onset or patient characteristics. The heterogeneity exists among the included studies. Although the random effects model allows for the presence of heterogeneity, there may still be some controversy about combining study estimates in its presence. Furthermore, as the low average age of patients (mainly in 30–40 s on average) was reported in many of the studies, the estimates for the prevalence of these disorders in older patients and children could not be analyzed because of the limited information obtained from the studied articles.

### 4.2 Future Directions

Olfactory and gustatory abnormalities have recently been reported in patients with COVID‐19, a global pandemic that we are still working tirelessly to contain. Our study validated recent reports by showing a high prevalence of these symptoms. Pathognomonic symptoms will help identify COVID‐19 positive patients who are otherwise asymptomatic and aid in narrowing a non‐specific respiratory diagnosis. Future analyses are needed to characterize the onset of taste/smell dysfunction, recovery rates, and the prevalence based on patient baseline characteristics and comorbidities.

## CONFLICT OF INTEREST

All authors have no COI to report.

## AUTHORS' CONTRIBUTION

Bahareh Hajikhani, Mohammad Javad Nasiri, Parnian Jamshidi, Masoud Dadashi, and Mehdi Goudarzi designed and performed the review literatures, collected the data, and helped in manuscript preparation. Tess Calcagno and Mehdi Mirsaeidi wrote the manuscript. Adrien Eshraghi critically reviewed the manuscript. All authors provided critical conceptual input and critically revised the report.

## ETHICAL STATEMENT

Dr. Mehdi Mirsaeidi is the U.S. federal employee and the opinions expressed in this article are the author's own and do not reflect Veteran Health Administration.
